# Impact of APOL1 polymorphism and IL-1β priming in the entry and persistence of HIV-1 in human podocytes

**DOI:** 10.1186/s12977-016-0296-3

**Published:** 2016-09-06

**Authors:** Joanna Mikulak, Ferdinando Oriolo, Federica Portale, Paolo Tentorio, Xiqian Lan, Moin A. Saleem, Karl Skorecki, Pravin C. Singhal, Domenico Mavilio

**Affiliations:** 1 Unit of Clinical and Experimental Immunology, Humanitas Clinical and Research Center, Rozzano, Milan Italy; 2 Istituto di Ricerca Genetica e Biomedica, UOS di Milano, Consiglio Nazionale delle Ricerche (UOS/IRGB/CNR), Rozzano, Milan Italy; 3 Center for Excellence for Immunology and Inflammation, Feinstein Institute for Medical Research, Hofstra North Shore Long Island Jewish Medical School, New Hyde Park, NY USA; 4 Children’s Renal Unit and Academic Renal Unit, University of Bristol, Bristol, UK; 5 Nephrology and Molecular Medicine, Technion Institute of Technology and Rambam Medical Center, Haifa, Israel; 6 Department of Medical Biotechnologies and Translational Medicine (BioMeTra), University of Milan, Milan, Italy

**Keywords:** APOL1, HIV-1, HIVAN, Podocyte, IL-1β, DC-SIGN

## Abstract

**Background:**

Patients of African ancestry with untreated HIV-1 infection and carrying the *G1* or *G2* kidney disease risk variants (Vs) at the *APOL1* gene have a tenfold higher risk of developing HIV-associated nephropathy (HIVAN) compared to those with the non-risk wild type (WT) G0 variant. However, the mechanistic contribution of the *APOL1* allelic state to kidney injury in HIV-1 infection remains to be elucidated.

**Results:**

Non-risk WT APOL1 is associated with lower intracellular levels of HIV-1 in conditionally immortalized human podocytes, while the over expression of G1 or G2 risk Vs significantly increases viral accumulation. The priming of podocytes with exogenous IL-1β facilitates HIV-1 entry, via the up-regulation of DC-SIGN. The over expression of APOL1 G1 and G2 risk Vs in combination with an increase in IL-1β levels causes a greater increase in viral concentration than either condition alone. In turn, HIV-1 and exogenous IL-1β together induce a de novo secretion of endogenous IL-1β and an increase of APOL1 gene expression.

**Conclusions:**

Our findings indicate that the presence of risk Vs of APOL1 is permissive of HIV-1 persistence in human podocytes in synergy with IL-1β, a cytokine that characterizes the inflammatory milieu of acute and chronic phases of HIV-1 infection. The elucidation of these molecular mechanisms explains, at least in part, the higher frequency of HIVAN in populations carrying the risk polymorphic genetic variant of *APOL1* gene.

## Background

HIV-1 associated nephropathy (HIVAN) is an important complication of HIV-1 infection and is characterized by collapsing focal segmental glomerulosclerosis (FSGS) and massive proteinuria [1, 2]. Different risk factors contribute to the pathogenesis of HIVAN. A longstanding high viral load is a major risk factor for the development of HIVAN [3–6]. Immune activation and chronic inflammation are additional risk factors for the onset of HIVAN together with specific genetic mutations in the host [7–10]. Indeed, a direct association between kidney risk variants (Vs) of the *Apolipoprotein L1* (*APOL1*) gene and the development of HIVAN had been reported [11–17]. These allelic Vs have been termed *G1* (*Ser342Gly and Ile384Met*) and *G2* (*del.N388/Y389*), in contrast to the non-risk wild type (WT) *G0* allele. APOL1 is a minor component of plasma circulating High-Density Lipoprotein (HDL) endowed with the ability to kill *Trypanosoma brucei* responsible for African sleeping sickness [18–22]. The emerging resistance to the non-risk WT allele by *Trypanosoma brucei gambiense and rhodesiense* remarkably increased the frequency of the risk Vs of APOL1 in the residents of many regions of Sub-Saharan Africa as a consequence of pathogen selection pressure [23]. Intracellular expression of APOL1 has been reported in several cell types, including podocytes, and appears to be a lipid-binding protein relevant for cellular homeostasis through endosomal trafficking regulation, for lysosomal function and autophagy ruling and for activation of innate immune response [24–28]. However, the molecular mechanisms explaining the role of APOL1 in the pathogenesis of HIVAN remain elusive.

Podocytes are epithelial cells acting in conjunction with fenestrated endothelium and glomerular basement membrane to ensure the integrity of the blood-urine barrier and glomerular filtration [29]. The challenge of studying human primary podocytes is due to their terminally differentiated phenotype. Development of the HIVAN transgenic murine model and the use of conditionally immortalized human podocytes (CIHPs) have provided in vivo and in vitro models that have greatly advanced our understanding of HIVAN physiopathology [30–32]. In particular, it has been demonstrated that viral gene products directly induce pathologic changes in the phenotype and functions of podocytes such as deregulations of several host cellular pathways that involve cell cycle, oxidative stress, and apoptosis [33–39]. Data from kidney biopsy of HIVAN patients also showed that podocytes host and accumulate HIV-1 and serve as viral reservoirs in kidney [4, 40]. Finally, in vitro experiments demonstrated that human podocytes are able to capture HIV-1 and spread the virus by trans-infecting target cells [41–43].

Interleukin-1β (IL-1β) possesses a strong pro-inflammatory effect. Its production is tightly controlled by two steps: (1) induction of *pro*-*IL*-*1*β gene expression and (2) caspase-1-mediated cleavage of pro-IL-1β through the activation of the inflammasome complexes [44]. Several types of viruses, including hepatitis C virus and HIV-1 have been found to induce the production of IL-1β through the induction of NLRP3 inflammasome [45, 46]. Recently, increased secretion of bioactive IL-1β was observed to be relevant for inflammatory programmed cell death (pyroptosis) of CD4^pos^ T cells with abortive HIV-1 infection [46]. Very little is known about the possible role of IL-1β in HIVAN pathogenesis.

Herein, we demonstrate that IL-1β in human podocytes facilitates HIV-1 trafficking by greatly enhancing the uptake of the virus via the up-regulation of DC-SIGN receptor. High intracellular levels of HIV-1 in synergy with IL-1β increase the expression of APOL1 that can serve as a natural anti-viral restriction factor. Indeed, the non-risk WT APOL1 gene product inhibits HIV-1 accumulation in podocyte, while the over expression of the APOL1 G1/G2 Vs increases the amount of HIV-1 in the same cells. These findings indicate that the allelic state of APOL1 is a key in determining the equilibrium between HIV-1 degradation and accumulation in human podocytes, thus representing a critical pathogenic factor in the pathogenesis of HIVAN.

## Results

### APOL1 targets HIV-1 trafficking in human podocytes

**Fig. 1:**
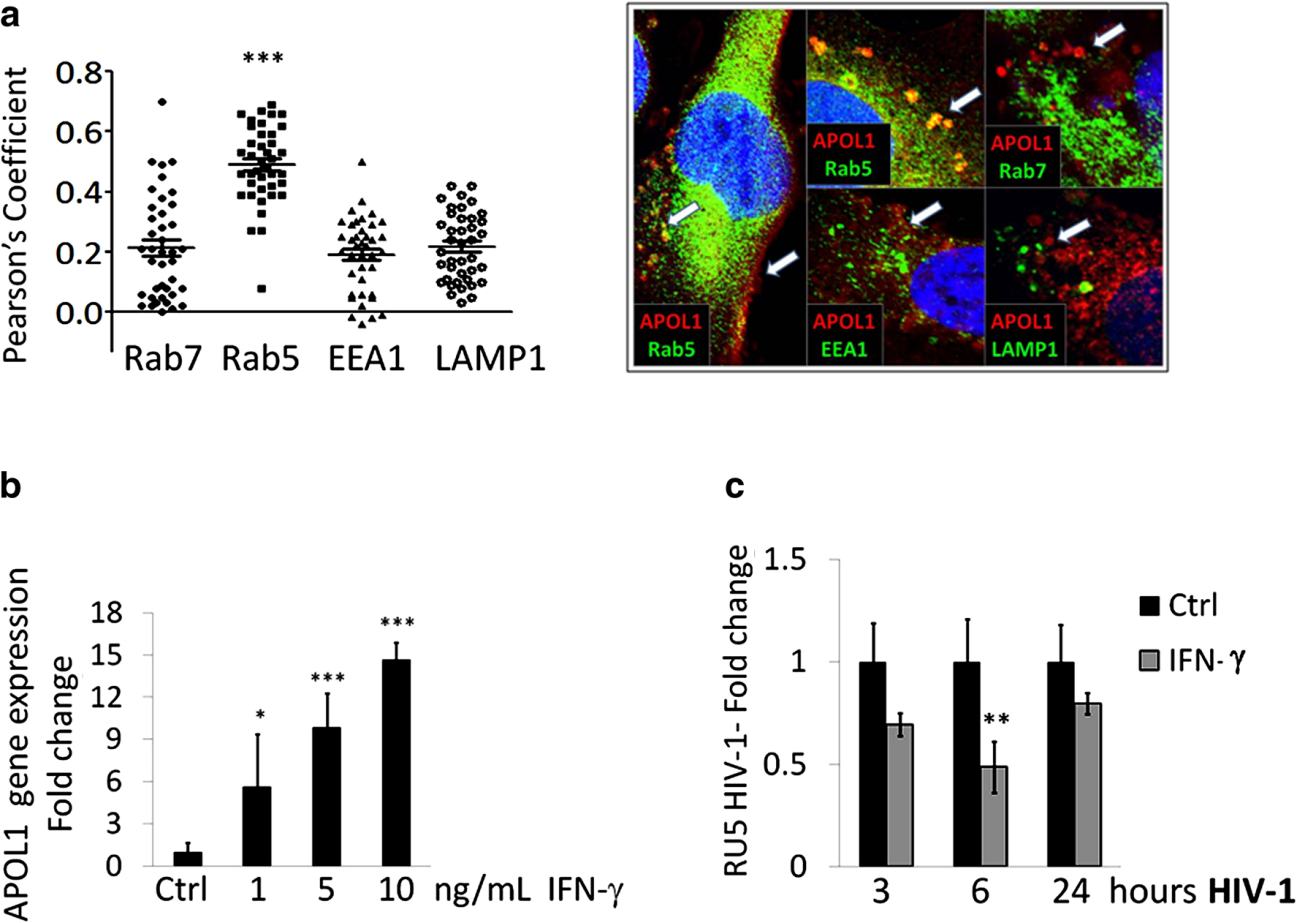
Increased expression of WT APOL1 inversely correlates to HIV-1 persistence in CIHPs. **a** Summary graph of statistical* dot plots* (*left panel*) showing the co-localization off WT APOL1 with Rab5, Rab7, EEA1 and LAMP1 as an average measured by Pearson’s Coefficient on data analyzed with the Olympus FLUOVIEW FV1000 confocal microscope software (*N* = 3). Representative fluorescent microscopic images (*right panel*) showing the co-localization of WT APOL1 (*red*) with Rab5, Rab7, EEA1 and LAMP1 (*green*) marked vesicles and plasma membrane in CIHPs. Nucleuses are stained with DAPI in *blue*. **b** Statistical histogram graph showing the dose-dependent response of APOL1 gene expression after IFN-γ stimulation (16 h). Results are expressed as relative fold change in IFN-γ treated CIHPs versus untreated controls (Ctrl) and normalized to GAPDH gene expression (*N* = 4). **c** Statistical histogram graph showing HIV-1 accumulation in CIHPs incubated for 16 h with IFN-γ (10 ng/mL) compared to their untreated counterparts (Ctrl). Results are expressed as relative fold change of HIV-1 DNA RU5 in IFN-γ treated cells versus Ctrl and normalized to GAPDH gene (*N* = 4). *P* values *<0.05, **<0.01; ***<0.001

It has been previously reported that human podocytes constitutively express APOL1 [26, 27]. Our experiments of confocal microscopy showed that APOL1 protein is located in proximity of the cellular membrane of CIHPs and preferentially co-localize with early endocytosed vesicles expressing Rab5 protein and not with endosomes marker EEA1 required for endosomes maturation. On the other hand, we did not observe APOL1 co-localization with either Rab7 or LAMP1 molecules which are expressed in late endosomes and lysosome compartments respectively (Fig. 1a). These findings prompted us to study a possible role of APOL1 in regulating HIV-1 entry and trafficking within human podocytes.

Since IFN-γ is a known inducer of APOL1 expression in podocytes [27, 28], CIHPs were pre-stimulated with IFN-γ. IFN-γ increases the APOL1 gene expression in CIHPs in a concentration-dependent manner (Fig. 1b). Previously it has been observed that in vitro experiments the capture of HIV-1 in human podocytes does not generate a productive virus replication [42, 43]. Therefore, to detect HIV-1 in podocytes we measured the specific HIV-1 strong stop DNA (RU5 HIV-1). This is possible because virions harbor strong-stop DNA since the endogenous reverse transcription of HIV-1 occurs prior to infection of target cells [47].

We then determined the viral entry in the podocytes by measuring the specific HIV-1 strong stop DNA concentration. Our data then demonstrated that the amount of HIV-1 significantly decreases in CIHPs treated with IFN-γ compared to their untreated counterparts (Fig. 1c). These results suggest that the IFN-γ-mediated up-regulation of APOL1 interfere with the entry and/or early post-entry steps regulating HIV-1 trafficking in podocytes. In this regard, we previously reported that HIV-1 internalization in CIHPs is mediated by both lipids raft and DC-SIGN receptor [41, 42]. However, IFN-γ treatment did not have any effect on DC-SIGN expression in podocytes (data not shown), thus ruling out a direct contribution of this inflammatory cytokine in limiting the viral entry in CIHPs through the modulation of this lectin-type receptor.

**Fig. 2:**
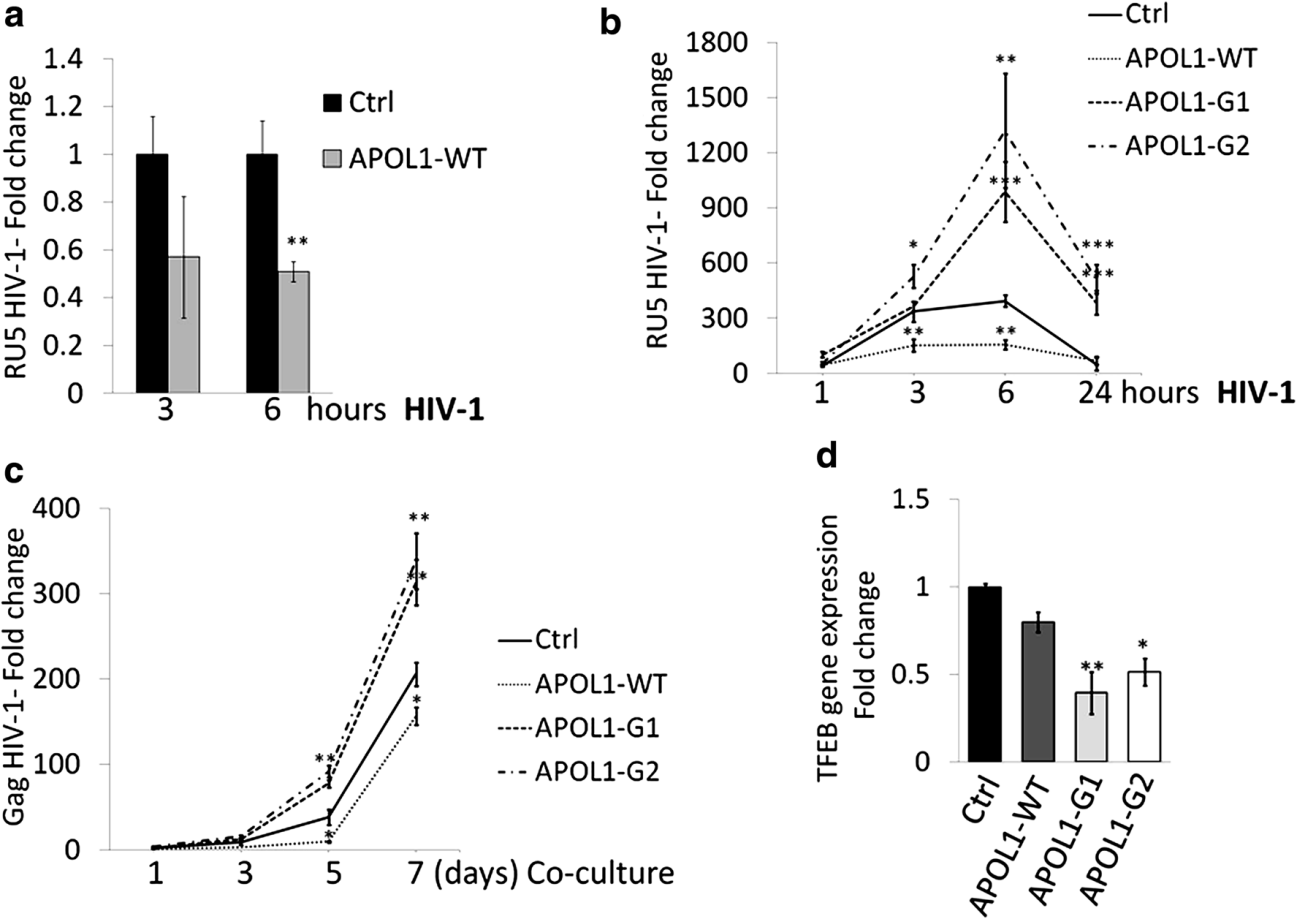
APOL1 polymorphism regulates HIV-1 persistence in CIHPs. **a** Statistical histogram graph showing HIV-1 accumulation in CIHPs transiently transfected with a APOL1-WT expressing vector compared to the control vector (Ctrl) following a time course incubation (3 and 6 h) with the virus. Results are expressed as relative fold change of HIV-1 DNA RU5 in APOL1-WT transfected cells compared to Ctrl and normalized to GAPDH gene (*N* = 4). **b** Time-course experiments showing the HIV-1 accumulation in CIHPs stable transfected with the control vector (Ctrl) or the specific APOL1-WT or APOL1-G1/G2 expressing vectors. Results are expressed as relative fold change of HIV-1 DNA RU5 in HIV-1 incubated CIHPs versus non HIV-1 treated cells and normalized to GAPDH gene (*N* = 4). **c** Rescue of infectious HIV-1 by CD4^pos^ T lymphocytes co-cultured in time-course experiments (1, 3, 5 and 7 days) with HIV-1 pulsed CIHPs stable transfected with APOL1-WT or APOL1-G1 or APOL1-G2 compared with cells transfected with control vector (Ctrl). Since HIV-1 internalization in human podocytes is characterized by an abortive HIV-1 infection, cell lysates were collected and analyzed by qPCR for HIV-1 Gag gene not present in podocytes and indicative of a productive infection in CD4^pos^ T cells. Results are expressed as fold increased of HIV-1 DNA Gag gene in PBMC co-cultured with HIV-1-pulsed CIHPs transfected with APOL1-WT or APOL1-G1 or APOL1-G2 or an empty vector (Ctrl) compared to their HIV-1 untreated counterparts and normalized to the amount of GAPDH gene (*N* = 4). **d** Statistical histogram graph showing TFEB gene expression in CIHPs transiently transfected with control vector (Ctrl) or APOL1-WT or APOL1-G1 or APOL1-G2 expressing vector. Results are expressed as fold change of transfected cells compared to Ctrl and normalized to the expression of GAPDH gene (*N* = 4). *P* values: *<0.05, **<0.01; ***<0.001

We then analyzed whether APOL1 plays a direct role in regulating HIV-1 accumulation in human podocytes. To this end, we transiently transfected CIHPs with the non-risk WT allele of APOL1. Similar to CIHPs stimulated with IFN-γ, we found that increased expression of WT APOL1 significantly decrease the levels of HIV-1 compared with CIHPs transfected with an empty control vector (Fig. 2a). We then proceeded to assess the effect of risk G1 and G2 APOL1 allelic Vs on HIV-1 accumulation in CIHPs stably transfected with either WT or G1/G2 APOL1 Vs. In contrast with non-risk WT APOL1, the over expression of the G1 or the G2 APOL1 Vs significantly increased HIV-1 levels in CIHPs (Fig. 2b), thus demonstrating that these risk Vs of APOL1facilitate HIV-1 accumulation and persistence in human podocytes. The higher amounts of virus in CIHPs transfected with the G1 or the G2 APOL1 Vs also induced a significant higher trans-infection of co-cultured CD4^pos^ T cells that showed a significant increase of viral replication compared to that exerted by CIHPs transfected with WT APOL1 allele (Fig. 2c).

We also assessed in podocytes the transcriptional levels of TFEB, the master transcription factor that regulates lysosome biosynthesis and whose activity is regulated by APOL1 expression [28, 48]. In addition, in human macrophages APOL1-dependent expression of TFEB was associated with high HIV-1 Gag protein degradation [28, 48]. Although, we did not observe differences in TFEB gene expression in WT APOL1 transfected podocytes, over expression of G1/G2 Vs of APOL1 significantly decrease the TFEB gene expression in CIHPs compared to that of cells transfected with WT APOL1 allele (Fig. 2d). These findings suggest that the APOL1 risk Vs may promote HIV-1 accumulation in human podocytes through the disruption of endosomal/lysosomal pathway regulation.

### IL-1β facilitates HIV-1 entry in human podocytes via DC-SIGN and increases viral accumulation

**Fig. 3:**
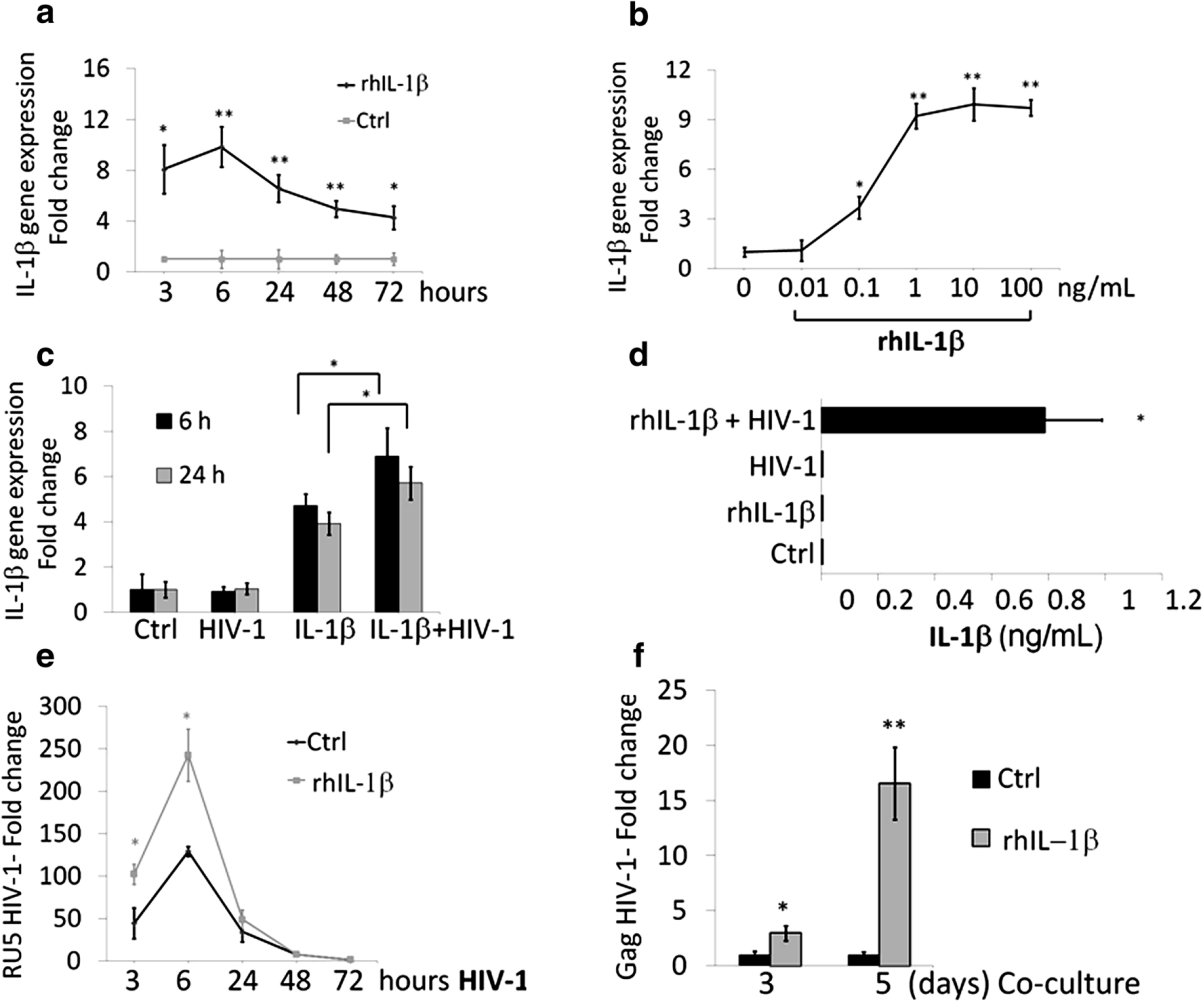
IL-1β increases HIV-1 accumulation in CIHPs. **a** Time and **b** dose response of IL-1β gene expression in unstimulated (Ctrl) and rhIL-1β treated CIHPs. Results are expressed as relative fold change in rhIL-1β treated cells versus Ctrl and normalized to GAPDH gene expression (*N* = 3). **c** Statistical histogram graph showing the IL-1β gene expression in CIHPs untreated (Ctrl), treated with rhIL-1β alone (25 ng/mL) for 16 h or incubated with HIV-1 either alone (6, 24 h) or with IL-1β (6, 24 h). Results are expressed as fold change of IL-1β gene expression in treated cells versus Ctrl and normalized to the GAPDH gene expression (*N* *=* 5). **d** Statistical histogram graph showing the amount of IL-1β protein secretion by CIHPs primed with rhIL-1β (25 ng/mL) and subsequently incubated with HIV-1 for 6 h compared to their counterparts incubated with rhIL-1β alone, HIV-1 alone or untreated cells (Ctrl) (*N* = 5). **e** Time course experiments showing HIV-1 accumulation in CIHPs either untreated (Ctrl) or pre-stimulated with rhIL-1β (25 ng/mL) for 16 h and followed by incubation with HIV-1 at different time points (3, 6, 24, 48, 72 h). Results are expressed as relative fold change of HIV-1 DNA RU5 in HIV-1 treated cells compared to their HIV-1 untreated counterparts for both experimental settings and normalized to GAPDH gene (*N* = 5). **f** Statistical histogram graph showing the rescue of infectious HIV-1 by CD4^pos^ T lymphocytes co-cultured for 3 and 5 days with HIV-1-pulsed CIHPs either primed or none (Ctrl) with rhIL-1β. Since HIV-1 internalization in human podocytes is characterized by an abortive HIV-1 infection, cell lysates were collected and analyzed by qPCR for HIV-1 Gag gene not present in podocytes and indicative of a productive infection in CD4^pos^ T cells. Results are expressed as relative fold change of DNA HIV-1 Gag copies in rhIL-1β treated compared to Ctrl and normalized to DNA copies of GAPDH gene (*N* = *5*). *P* values: *<0.05, **<0.01

The establishment of inflammation together with the pathologic production of several pro-inflammatory cytokines is a hallmark of HIV-1 infection. In particular, the increased systemic levels of IL-1β have been found in the sera of HIV-1 infected patients both in acute and chronic phases of the disease [49–52]. More recently, it has also been reported that abortive infection of HIV-1 in CD4^pos^ T cells induces an inflammatory programmed cell death (i.e. pyroptosis) caused by the release of pro-inflammatory cytokines including IL-1β [46]. Furthermore, an increased expression of inflammasome markers such as IL-1β and caspases-1 was observed in the renal cortex of HIV-transgenic mice (Tg26) [53]. This experimental evidence prompted us to analyze the direct effect of exogenous rhIL-1β on CIHPs at different time points. We found that the stimulation of CIHPs with 25 ng/mL of rhIL-1β induce a rapid and significant increase of *IL*-*1*β gene expression that reached a plateau after 6 h of incubation (Fig. 3a). This phenomenon appeared to be also dose-dependent as the maximum response was reached at the concentration of 1.0 ng/mL of rhIL-1β (Fig. 3b). Moreover, the incubation of rhIL-1β-primed CIHPs with HIV-1 also induced a significantly higher IL-1β transcription levels compare to CIHPs stimulated with rhIL-1β alone (Fig. 3c). Nevertheless, the treatment of CIHPs with HIV-1 alone did not induce any increase of the IL-1β gene expression nor any release of detectable levels of IL-1β in cell supernatant (Fig. 3d). Only the stimulation of podocytes with rhIL-1β followed by incubation with HIV-1 induced a detectable secretion of endogenous IL-1β (Fig. 3d). These data suggest that increased levels of circulating IL-1β in the glomerular milieu of HIV-1 infected patients can induce a pathologic inflammatory loop that leads to the production and secretion of endogenous IL-1β by human podocytes.

**Fig. 4:**
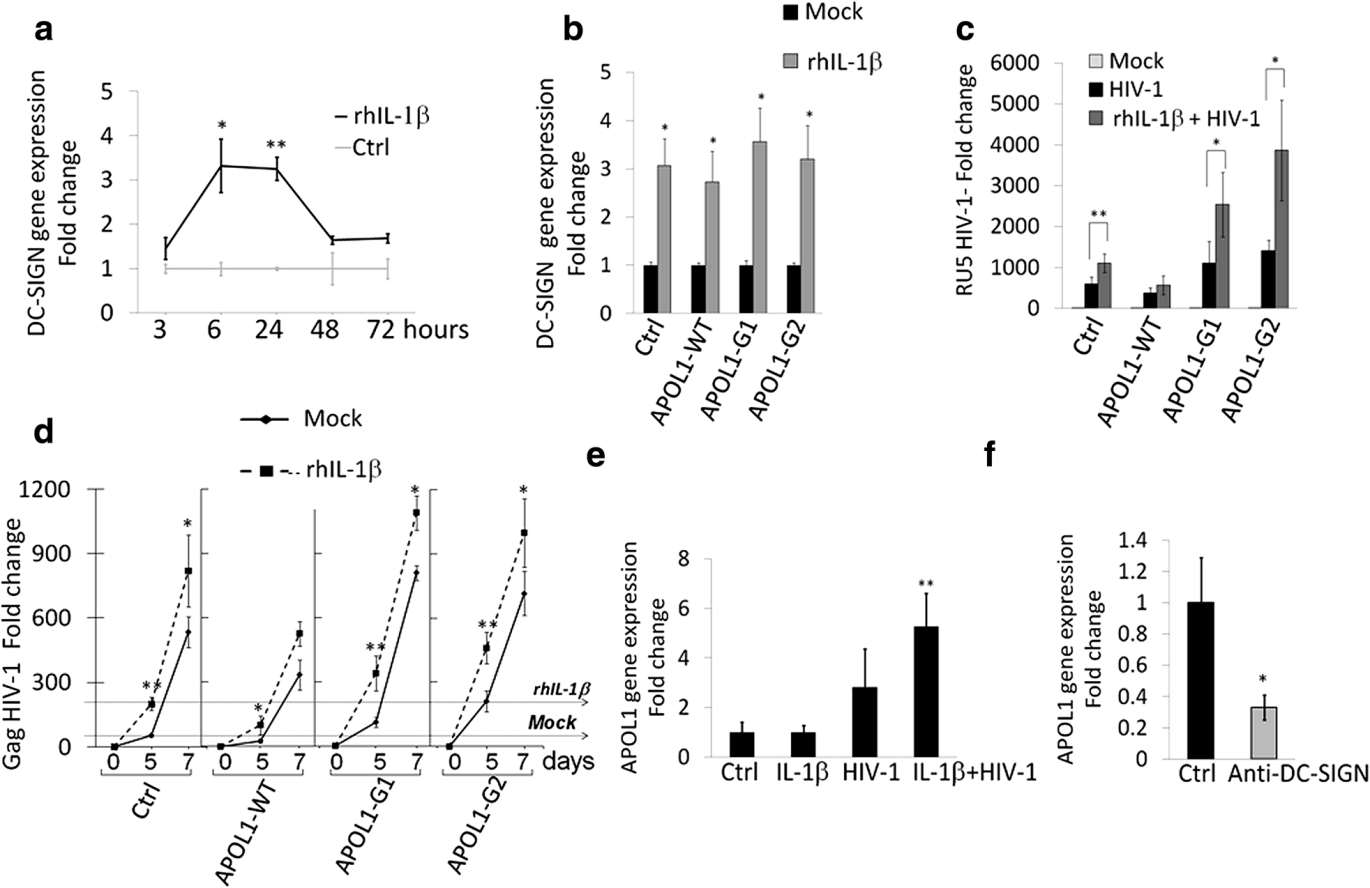
APOL1 polymorphism affects IL-1β-dependent persistence of HIV-1 in CIHPs. **a** Time course experiments showing DC-SIGN gene expression in CIHPs after stimulation with rhIL-1β (25 ng/mL) compared to untreated cells (Ctrl) and normalized to GAPDH gene expression (*N* = 3). **b** Statistical histogram graph showing DC-SIGN gene expression after 18 h of rhIL-1β (25 ng/mL) treatment in CIHPs stably transfected with either empty vector (Ctrl) or WT or APOL1 risk Vs. Results are expressed as relative fold change in rhIL-1β treated cells versus non treated cells (Mock) and normalized to GAPDH gene expression (*N* = 3). **c** Statistical histogram graph showing HIV-1 accumulation after rhIL-1β stimulation in CIHPs stably transfected with the control vector (Ctrl) or the specific APOL1-WT or APOL1-G1/G2 expressing vectors. Results are expressed as relative fold change of HIV-1 DNA RU5 in HIV-1 incubated CIHPs versus non HIV-1-pulsed cells (Mock) and normalized to GAPDH gene (*N* = 3). **d** Rescue of infectious HIV-1 by CD4^pos^ T lymphocytes co-cultured in time-course experiments (0, 5 and 7 days) with HIV-1 pulsed CIHPs stable transfected with control vector (Ctrl), APOL1-WT or APOL1-G1/G2 Vs and pretreated or not (Mock) with rhIL-1β. Results are expressed as fold increased of HIV-1 DNA Gag gene in PBMC co-cultured with HIV-1 pulsed CIHPs compared to their HIV-1 untreated counterparts and normalized to the amount of GAPDH gene (*N* = 3). Horizontal lines represent *Mock* and *rhIL*-*1*β in the Ctrl sample depicting the fold change in HIV-1 replication. **e** APOL1 gene expression in CIHPs incubated with rhIL-1β (25 ng/mL) and subsequently treated with HIV-1 for 6 h compared to their counterparts incubated with rhIL-1β alone, HIV-1 alone or untreated (Ctrl) Results are expressed as relative fold change normalized to GAPDH gene expression (*N* = 5). **f** Statistical histogram graph showing the APOL1 gene expression in CIHPs incubated with IL-1β (25 ng/mL) and subsequently treated with HIV-1 for 6 h either in pre absence (Ctrl) or in the presence of blocking anti-DC-SIGN mAb. Results are expressed as relative fold change in treated cells normalized to GAPDH gene expression (*N* = 3). *P* values *< 0.05, **< 0.01

We then assessed the effect of rhIL-1β in viral trafficking within human podocytes by stimulating CIHPs with 25 ng/mL of rhIL-1β for 16 h followed by incubation with HIV-1. Cells were harvested at different time-points and HIV-1 strong stop DNA was measured by qPCR. The priming of CIHPs with rhIL-1β significantly increased the amount of HIV-1 compared to podocytes incubated in the absence of rhIL-1β (Fig. 3e). The higher concentration of HIV-1 in rhIL-1β-primed CIHPs also induced a significantly higher trans-infection of co-cultured CD4^pos^ T lymphocytes that showed an increase of viral replication compared to that exerted by CIHPs not primed with rhIL-1β (Fig. 3f). Given that HIV-1 entry in CIHPs depends on DC-SIGN expression [42] we than analyzed the transcripts levels of DC-SIGN in podocytes following stimulation with rhIL-1β. The incubation with rhIL-1β induced a rapid and significant increase of DC-SIGN gene expression in CIHPs (Fig. 4a). These results indicate that the inflammatory loop triggered by rhIL-1β is permissive of HIV-1 internalization in CIHPs via the up-regulation of DC-SIGN, which represent an already reported mechanism employed by HIV-1 to enter in these cells [42].

RhIL-1β increases expression of DC-SIGN also in CIHPs stably transfected with either WT or G1/G2 APOL1 Vs (Fig. 4b), therefore, we used this experimental model to test the direct impact of rhIL-1β priming on HIV-1 entry and trans-infection to CD4^pos^ T lymphocytes in the context of APOL1 polymorphism. RhIL-1β priming in both G1 and G2 APOL1 risk Vs was associated with the significantly increased accumulation of HIV-1. This phenomenon was correlated with the higher trans-infection of CD4^pos^ T lymphocytes compared to the non treated cells with rhIL-1β (Fig. 4c). Podocytes transfected with APOL1 WT and pre-treated with rhIL-1β also resulted with the significant upper trans-infection of CD4^pos^ T lymphocytes after 5 days of co-culture (Fig. 4d). Nevertheless, over expression of APOL WT in rhIL-1β pre-stimulated cells showed lower virus concentration versus rhIL-1β-primed CIHPs transfected with empty vector. These results confirmed that only the presence of APOL1 WT, and not of APOL1 Vs, is associated with the control of HIV-1 entry and accumulation in podocytes.

In addition, we found that the incubation of CIHPs with rhIL-1β and HIV-1 together is required to raise the level of APOL1 gene expression, as neither rhIL-1β nor HIV-1 alone induced a significant increase of its transcript when compared to control experiments (Fig. 4e). To verify whether the up-regulation of APOL1 expression in rhIL-1β-primed CIHPs is dependent on HIV-1 binding to DC-SIGN receptor, cells were pre-treated with a masking anti-DC-SIGN mAb before incubation with HIV-1. We found that the blocking of DC-SIGN induced a significant decrease of APOL1 gene expression, thus reversing the up-regulation of this apolipoprotein in response to simultaneous stimulation given by rhIL-1β and HIV-1 (Fig. 4f).

## Discussion

**Fig. 5:**
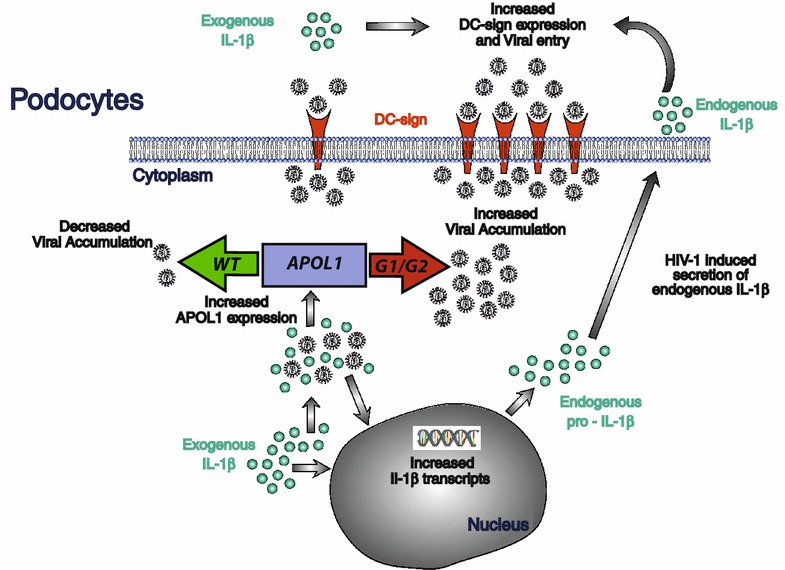
Mechanistic insights regulating HIV-1 entry and persistence in human podocytes. High levels of IL-1β both in tissues and in blood characterize acute and chronic HIV-1 infection and can prime human podocytes in the context of the inflamed microenvironment of kidney glomerula. Indeed, exogenous IL-1β enhances HIV-1 entry via the up-regulation of DC SIGN and induces the production/secretion of endogenous IL-1β. In turn, the presence of both IL-1β and HIV-1 increase the expression of APOL1 within podocytes. In this context, the non-risk WT variant of APOL1 is key to control viral accumulation within podocytes and limit HIV-1 boarding. On the other side, the alternative presence of the high-risk G1 or G2 polymorphic variants of APOL1 increase the accumulation of HIV-1 in podocytes, thus worsening the vicious pathogenic inflammatory loop in podocytes. This can explain, at least in part, the higher frequency of HIVAN in populations carrying the high-risk genetic variants of *APOL1* gene

The pathogenic role of APOL1 is emerging in several kidney diseases targeting podocytes and strong association between APOL1 high-risk Vs and progressive non-diabetic kidney disease [15, 26, 54], FSGS and HIVAN [11–17] have been reported. The present study demonstrates that risk G1 and G2 variants of APOL1 enhances HIV-1 accumulation and persistence in human podocytes, and also contributes to spread infection to neighbor CD4^pos^ T cells. This process is amplified by IL-1β, whose priming of human podocytes increases viral entry and, together with HIV-1 itself, up-regulates the APOL1 gene expression (Fig. 5).

Previous in vitro studies reported that the over expression of APOL1 in podocytes induces lysosomal swelling and cell death with the G1 and G2 risk variants exerting stronger effects at a lower threshold compared to the non-risk WT APOL1 [55]. The present study first sought to examine the impact of *APOL1* polymorphism in regulating HIV-1 trafficking in human podocytes. Indeed, these cells are able to capture HIV-1 that, instead of fully replicating to generate a productive infection, is rapidly degraded [42, 43]. We demonstrate here that the transfection of non-risk WT APOL1 allele in CIHPs decreases the intracellular concentration of HIV-1, thus highlighting that the WT variant of APOL1 serves as a natural anti-viral restriction factor in podocytes. Our additional experimental evidence showing that the expression of APOL1 is increased in rhIL-1β-primed podocytes following HIV-1 entry further supports this hypothesis. In this regard, it has been also shown that the anti-viral cytokine IFN-γ is able to greatly increase the expression of APOL1 [27] that, in turn, can target multiple steps of HIV-1 replication cycle in monocytes with the final result of inducing viral endocytosis and degradation within lysosome compartments [28]. Our data confirmed that also the stimulation of CIHPs with IFN-γ induce higher transcription levels of APOL1 that, as an intracellular protein, co-localize with Rab5 early endosomes. Of note, it has been already reported that Rab5 early endosomes regulate the homeostasis of endosomal pathway of podocytes [56], and that binding of HIV-1 to DC-SIGN leads to an internalization of the virus in early Rab5 positive endosomes [57]. These findings suggest that the IFN-γ-mediated up-regulation of APOL1, together with DC-SIGN-mediated uptake of the virus, likely play a major role in endocytic trafficking and degradation of HIV-1 also in human podocytes.

Another important contribution to antiviral responses of podocytes might occur as a consequence of the inflammatory processes that characterize both acute and chronic HIV-1 infection. In particular, both circulating and tissue resident immune cells (i.e. monocytes/macrophages, T CD4^pos^ lymphocytes, gut epithelial cells, etc.) have been reported to produce high amount of IL-1β that, in turn, boost local and systemic inflammation during the course of HIV-1 disease [46, 50, 58–60]. Indeed, high levels of IL-1β have been found in the sera of HIV-1 infected patients [61–63]. Although the harmful effect of this potent cytokine in increasing viral replication and in directly damaging several immune and not immune cells both in blood and tissue have been extensively reported [46, 59, 60, 64–66], little is known about the pathogenic role of IL-1β in HIV-1 targeted podocytes. Recently, it has been shown in a murine HIVAN model (Tg26) that HIV-1 induces the activation of the inflammasome in podocytes [53]. We show here that the stimulation of podocytes with exogenous rhIL-1β induces a de novo IL-1β gene transcription. However, the secretion of bioactive endogenous IL-1β requires a second signal delivered by HIV-1 following its uptake and internalization in podocytes. In this regard, we already reported the HIV-1 entry in human podocytes is mainly mediated by DC-SIGN [42], a lectin-type surface receptor able to bind HIV-1 in a CD4-independent manner and promote trans-infection to CD4^pos^ T cells [67]. Additionally, we also demonstrate here that in vitro stimulation of CIHPs with rhIL-1β increases the expression of DC-SIGN, thus explaining both the higher accumulation of HIV-1 in rhIL-1β-primed podocytes and the increased trans-infection of co-cultured CD4^pos^ T cells. Hence, the priming with rhIL-1β facilitates viral entry into podocytes and triggers a harmful and vicious loop that increases viral spreading and induces a de novo secretion of endogenous IL-1β by podocytes. In this regard, it has also been reported that under inflammatory stress podocytes can serve as non-hematopoietic professional antigen-presenting cells that up-regulate the expression of MHC class-II molecules [68–70]. The elucidation of these mechanisms in the context of the inflamed glomerular milieu of HIV-1 infected patients containing high levels of IL-1β help us to better understand the pathological changes observed in kidneys that ultimately culminate in cell injuries and organ failure [36–39].

In the context of the HIV-1 driven inflammation in kidney, we demonstrate that the non-risk WT variant of APOL1 is endowed with natural antiviral activity that limits HIV-1 accumulation in podocytes. Indeed, our data showing that rhIL-1β priming in synergy with HIV-1 increases the gene expression of this apolipoprotein in podocytes via the up-regulation of DC-SIGN explain, at least in part, the low incidence of HIVAN in the general population carrying the WT APOL1. This is not the case for those populations carrying the risk G1 and G2 polymorphic variants of APOL1 that, following HIV-1 infection, experience a much higher frequency of HIVAN development [11–17]. The present study demonstrates that the over expression of these latter risk Vs of APOL1 is associated with significantly higher HIV-1 accumulation in podocytes, thus representing a relevant risk factor for the onset of HIVAN. Furthermore, the expression of APOL1 G1 and G2 risk Vs in combination with an increase in IL-1β levels causes a greater increase in viral concentration than either condition alone. Since, the risk Vs of APOL1 inhibit in podocytes the gene expression of TFEB, a master transcription factor that regulates lysosome biosynthesis [48], this strongly suggests that the increased boarding of HIV-1 in the podocytes carrying the G1 or G2 APOL1 polymorphism is due to a defective viral clearance. Furthermore, it has been previously reported that only APOL1 risk Vs could induce lysosomal leakage in podocytes effects at a lower threshold compared to the non-risk WT APOL1 [55]. The reversal from clearance to enhanced boarding of HIV-1 as a function of the non-risk WT versus risk G1 or G2 alleles state of APOL1, may explain why, of all podocytopathic kidney diseases, HIVAN shows the most striking odds ratio, reading >89 in one recent study [71].

## Conclusions

The present study demonstrates that APOL1 risk polymorphic Vs have a great impact in increasing uptake, accumulation and persistence of the HIV-1 in human podocytes. The inflammatory milieu that characterizes HIV-1 infection both at systemic and tissue levels can enhance this pathologic loop via the IL-1β priming. This explains, at least in part, the higher frequency of HIVAN in populations carrying the G1 and G2 genetic variants of *APOL1* gene (Fig. 5).

## Methods

### Cells and virus

Conditionally immortalized human podocytes (CIHPs) were developed and cultured as described in Saleem et al. [30]. CIHPs were transiently transfected with the empty vector or pcDNA3-APOL1-WT or -APOL1-G1/G2 expressing plasmid by using as a transfection reagent Lipofectamine 2000 (Invitrogen, Carlsbad, CA, USA) according to the manufacturer’s instructions. Meanwhile 48–72 h following transfection cells were used for qPCR analysis or treated with HIV-1_BaL_. Stable G0/G1/G2 APOL1 CIHPs were generated by retroviral infection. Briefly, the open reading frame APOL1 (G0, G1, G2) was cloned into the retroviral vector pBABE carrying resistance to puromycin. To generate retroviral particles, the viral packaging cell line HEK-GP were co-transfected with the pBABE construct of interest and the VSV gene. CIHPs were infected twice within 24 h with the viral-containing supernatant of HEK-GP cells. Selection with puromycin (1 μg/mL) was continued for a week, and comparable expression of respective sequence of the corresponding APOL1 was verified.

Human PBMCs were obtain from Buffy coats of healthy volunteers who signed consent forms in accordance with the Declaration of Helsinki and with clinical protocols approved by the Institutional Review Board of Desio Hospital, Milan, Italy. PBMCs were isolated over Ficoll Paque™ Premium density gradients (GE Healthcare Bio-Sciences AB, Little Chalfont, Buckinghamshire, UK) and cultured in RPMI 1640 medium, supplemented with 10 % FBS, 2 mM l-glutamine, 100 U/mL penicillin/streptomycin and activated with 5 μg/mL of Lectin from *Phaseolus vulgaris* (PHA-L) (Sigma-Aldrich, Saint Louis, MO, USA) for 3 days PHA-L and subsequently with 200 IU/mL of human recombinant IL-2 (Peprotech, Rocky Hill, NJ, USA). In co-cultivation studies activated lymphocytes were added to podocytes at a ratio of 5:1 in a RPMI complete medium supplemented with 200 U/mL of IL-2. HIV-1_BAL_ strain was expanded in activated PBMCs collected at the peak of virus replication. HIV-1 p24 Ag concentrations in the culture supernatants were determined by ELISA (Aalto, Ratfharnham Village, Ireland) as described in [42].

### Reagents

Human recombinant (h) IL-1β and interferon-γ were purchased from PeproTech. Human IL-1β secreted protein was measured in cell-free harvested supernatants by ELISA (DuoSet DY201, R&D System, Minneapolis, MN, USA). In blocking experiments CIHPs were pre-treated with rIL-1β, washed out and then incubated with control Isotype (Ctrl) or mouse anti-DC-SIGN monoclonal Ab (mAb) at the dose of 5 mg/mL (Abcam) before HIV-1 treatment.

### QPCR

For cellular gene expression total RNA was extracted using RNeasy mini columns (Qiagen, Valencia, CA, USA), following manufacturer’s instructions. One μg of total RNA was used to generate cDNA templates for RT-PCR, using random primers, RNase inhibitor and High-Capacity cDNA Reverse Transcription Kit from Applied Biosystem (Foster City, CA, USA). IL-1β, TFEB, DC-SIGN, WT-1, podocin, nephrin, synaptopodin and housekeeping S18 and GAPDH genes expression were analyzed by the TaqMan^®^ mRNA specific assays. Followed primers were used for the SYBR Green PCR Master Mix analysis (Applied Biosystem): APOL1 Fw-5′-ATCTCAGCTGAAAGCGGTGAAC-3′ and Rev-5′-TGACTTTGCCCCCTCATGTAAG-3′; 18S Fw-5′-ACTTTCGATGGTAGTCGCCGT-3′ and Rev-5′-CCTTGGATGTGGTAGCCGTTT-3′.

### HIV-1 detection

Viral stock before incubation with podocytes were treated (1 h at room temperature) with 200 U/mL of RNase-free DNase (Roche, Basel, Switzerland). Before preparation of cell lysates, control and HIV-1-pulsed cells were washed three times with PBS, then treated with 0.05 % trypsin at 37 °C for 10 min to eliminate non internalized virus, and subsequently washed five times with PBS. Cell lysates were prepared as described in Malnati et al. [72] and subjected to qPCR TaqMan analysis by using HIV-1 specific primers: Gag Fw-5′-ACATCAAGCAGCCATGCAAAT-3′, Rev-5′-ATCTGGCCTGGTGCAATAGG-3′ and probe 5′-CATCAATGAGGAAGCTGCAGAATGGGATAGA-3′, LTR RU5 Fw-5′-GGCTAACTAGGGAACCCACTG-3′, Rev-5′-CTGCTAGAGATTTTCCACACTGAC-3′ and probe 5′-TGTGTGCCCGTCTGTTGTGTG-3′ [42, 73]. Amplification of GAPDH gene as a reference was used to control the amount of DNA in each sample (Applied Biosystem).

### Fluorescence confocal microscopy

CIHPs were growth on the coverslip’s coated with human collagen IV then were fixed with 4 % paraformaldehyde, followed by permeabilization and blocking with 0.3 % Triton X-100 and 10 % goat serum. Immunoblotting with the followed primary antibodies were performed: Mouse monoclonal anti-APOL1, anti-DC-SIGN and rabbit polyclonal anti-podocin purchased from Abcam; Rabbit polyclonal anti-Rab5, -Rab7, -EEA, -Lamp1 purchased from Life Technolgies.

### Statistical analysis

The significance of the data was assessed using Student’s *t* test statistical analysis. Data shown are mean ± S.D. The number of experiments is specified in the Figure legends. In the figures statistical significance (*P*) is indicated by asterisks (*). *P < 0.05; **P < 0.01; ***P < 0.001.
